# Supplementary material for: Comparative genomics provides new insights into the diversity, physiology, and sexuality of the only industrially exploited tremellomycete: Phaffia rhodozyma
Source: BMC Genomics. 2016 Nov 9;17:901. doi: 10.1186/s12864-016-3244-7 (PMC5103461; doi:10.1186/s12864-016-3244-7)
Supplement: Additional file 6: — List of orphan genes with links to PFAM (related to Additional file 1: Table S1). (ZIP 1428 kb) [file 12864_2016_3244_MOESM6_ESM.zip › BLAST_HTML_FTR/G00969_P.html]

BLAST Search Results


```
BLASTP 2.2.27+


Reference:
Stephen F. Altschul, Thomas L. Madden, Alejandro A. Schäffer,
Jinghui Zhang, Zheng Zhang, Webb Miller, and David J. Lipman (1997),
"Gapped BLAST and PSI-BLAST: a new generation of protein database
search programs", Nucleic Acids Res. 25:3389-3402.


Reference for
composition-based statistics:
Alejandro A. Schäffer, L. Aravind, Thomas L. Madden, Sergei
Shavirin, John L. Spouge, Yuri I. Wolf, Eugene V. Koonin, and
Stephen F. Altschul (2001), "Improving the accuracy of PSI-BLAST
protein database searches with composition-based statistics and
other refinements", Nucleic Acids Res. 29:2994-3005.


Database: nr
           71,551,133 sequences; 26,053,659,533 total letters


Query= G00969_P

Length=99
                                                                      Score     E
Sequences producing significant alignments:                          (Bits)  Value

emb|CED84764.1|  hypothetical protein [Xanthophyllomyces dendrorh...   188    2e-59
gb|KIJ17590.1|  hypothetical protein PAXINDRAFT_72185 [Paxillus i...  39.3    0.044
gb|KIJ64591.1|  hypothetical protein HYDPIDRAFT_90092 [Hydnomerul...  37.4    0.20 
gb|KIL00223.1|  hypothetical protein PAXRUDRAFT_130403 [Paxillus ...  35.4    1.1  
ref|XP_002486941.1|  conserved hypothetical protein [Talaromyces ...  35.4    1.4  
ref|XP_009265840.1|  Pre-mRNA polyadenylation factor fip1 [Wallem...  37.0    1.5  
gb|KDB18900.1|  SH3 domain-containing protein [Ustilaginoidea vir...  37.0    1.5  
dbj|GAO16745.1|  hypothetical protein UVI_040550 [Ustilaginoidea ...  36.6    2.5  
ref|XP_010156447.1|  PREDICTED: carboxypeptidase N subunit 2-like...  36.2    2.8  
ref|XP_007702317.1|  hypothetical protein COCSADRAFT_162491 [Bipo...  35.8    5.3  
ref|XP_007688806.1|  hypothetical protein COCMIDRAFT_97608 [Bipol...  35.4    6.8  
ref|WP_049326742.1|  membrane protein [Rothia mucilaginosa]           34.7    7.5  
ref|XP_007710260.1|  hypothetical protein COCCADRAFT_90853 [Bipol...  35.0    7.6  
gb|KIW96195.1|  hypothetical protein Z519_03263 [Cladophialophora...  35.0    8.5  


 >emb|CED84764.1| hypothetical protein [Xanthophyllomyces dendrorhous]
Length=96

 Score =  188 bits (478),  Expect = 2e-59, Method: Compositional matrix adjust.
 Identities = 95/95 (100%), Positives = 95/95 (100%), Gaps = 0/95 (0%)

Query  1   MSTAVSSTTYPAAAVTLDKKAMLPEVDIRREQPQVAPTMQSSSIPLSSTPVPSPPQVAQS  60
           MSTAVSSTTYPAAAVTLDKKAMLPEVDIRREQPQVAPTMQSSSIPLSSTPVPSPPQVAQS
Sbjct  1   MSTAVSSTTYPAAAVTLDKKAMLPEVDIRREQPQVAPTMQSSSIPLSSTPVPSPPQVAQS  60

Query  61  TGELEEAARIRGGGCCGACCGCFFGAIAAVLCCGL  95
           TGELEEAARIRGGGCCGACCGCFFGAIAAVLCCGL
Sbjct  61  TGELEEAARIRGGGCCGACCGCFFGAIAAVLCCGL  95


>gb|KIJ17590.1| hypothetical protein PAXINDRAFT_72185 [Paxillus involutus ATCC 
200175]
Length=69

 Score = 39.3 bits (90),  Expect = 0.044, Method: Compositional matrix adjust.
 Identities = 29/73 (40%), Positives = 33/73 (45%), Gaps = 11/73 (15%)

Query  22  MLPEVDIRREQPQVAPTMQSSSIPLSSTPVPSPPQVAQSTGELEEAARIRGGGCCGACCG  81
           M P       QPQ A  MQ S +       P+P   AQ+  E   A RIRGGG   A   
Sbjct  1   MEPTNKTTTNQPQAA--MQMSVVN------PTPGNNAQNHQEHHHAKRIRGGG---AAKD  49

Query  82  CFFGAIAAVLCCG  94
           CF GA+   LC G
Sbjct  50  CFLGALECFLCFG  62


>gb|KIJ64591.1| hypothetical protein HYDPIDRAFT_90092 [Hydnomerulius pinastri 
MD-312]
Length=69

 Score = 37.4 bits (85),  Expect = 0.20, Method: Compositional matrix adjust.
 Identities = 26/63 (41%), Positives = 30/63 (48%), Gaps = 11/63 (17%)

Query  32  QPQVAPTMQSSSIPLSSTPVPSPPQVAQSTGELEEAARIRGGGCCGACCGCFFGAIAAVL  91
           QPQ A  M        S   P+P   AQ+  E + A RIRGG   GA   CF GA+   L
Sbjct  11  QPQAAMQM--------SVMNPAPANNAQNHQEHQHAKRIRGG---GAGKDCFLGALECFL  59

Query  92  CCG  94
           C G
Sbjct  60  CFG  62


>gb|KIL00223.1| hypothetical protein PAXRUDRAFT_130403 [Paxillus rubicundulus 
Ve08.2h10]
Length=69

 Score = 35.4 bits (80),  Expect = 1.1, Method: Compositional matrix adjust.
 Identities = 28/73 (38%), Positives = 31/73 (42%), Gaps = 11/73 (15%)

Query  22  MLPEVDIRREQPQVAPTMQSSSIPLSSTPVPSPPQVAQSTGELEEAARIRGGGCCGACCG  81
           M P       QPQ A  MQ S +       P+P   AQ   E   A RIRGGG       
Sbjct  1   MDPINKTTTNQPQAA--MQMSVVS------PAPGNSAQDHQEHHRAKRIRGGGVAK---D  49

Query  82  CFFGAIAAVLCCG  94
           CF GA+   LC G
Sbjct  50  CFLGALECFLCFG  62


>ref|XP_002486941.1| conserved hypothetical protein [Talaromyces stipitatus ATCC 10500]
 gb|EED12830.1| conserved hypothetical protein [Talaromyces stipitatus ATCC 10500]
Length=84

 Score = 35.4 bits (80),  Expect = 1.4, Method: Compositional matrix adjust.
 Identities = 21/62 (34%), Positives = 33/62 (53%), Gaps = 2/62 (3%)

Query  29  RREQPQVA-PTMQSSSIPLSSTPVPSPPQVAQSTGELEEAARIRGG-GCCGACCGCFFGA  86
           + E+P+   PT   S++ +      +  Q A  + + E+  ++RGG GCC  CCGCF G 
Sbjct  15  QEEEPRTQQPTWNPSTMAMEQPANYAGSQQAAPSSQEEQQMKLRGGAGCCDVCCGCFAGL  74

Query  87  IA  88
            A
Sbjct  75  CA  76


>ref|XP_009265840.1| Pre-mRNA polyadenylation factor fip1 [Wallemia ichthyophaga EXF-994]
 gb|EOR04821.1| Pre-mRNA polyadenylation factor fip1 [Wallemia ichthyophaga EXF-994]
Length=577

 Score = 37.0 bits (84),  Expect = 1.5, Method: Composition-based stats.
 Identities = 23/56 (41%), Positives = 31/56 (55%), Gaps = 4/56 (7%)

Query  23   LPEVDIRREQ-PQVAPTMQSSSIPL---SSTPVPSPPQVAQSTGELEEAARIRGGG  74
            LP+ +I  E  PQ  P+  +S+ P+   S TPVP PP V  +T  L  AA + G G
Sbjct  111  LPKPNITTEYIPQERPSAVTSTPPVKAPSHTPVPQPPTVVDNTIALPSAANVAGTG  166


>gb|KDB18900.1| SH3 domain-containing protein [Ustilaginoidea virens]
Length=571

 Score = 37.0 bits (84),  Expect = 1.5, Method: Compositional matrix adjust.
 Identities = 20/51 (39%), Positives = 23/51 (45%), Gaps = 0/51 (0%)

Query  46   LSSTPVPSPPQVAQSTGELEEAARIRGGGCCGACCGCFFGAIAAVLCCGLV  96
            LSSTP P P   A S  +   A     G   GA  G  FG +  VL  GL+
Sbjct  142  LSSTPTPGPNPTATSVSDPSTATDTGDGASAGAKAGIAFGILGGVLIVGLI  192


>dbj|GAO16745.1| hypothetical protein UVI_040550 [Ustilaginoidea virens]
Length=551

 Score = 36.6 bits (83),  Expect = 2.5, Method: Compositional matrix adjust.
 Identities = 20/51 (39%), Positives = 23/51 (45%), Gaps = 0/51 (0%)

Query  46   LSSTPVPSPPQVAQSTGELEEAARIRGGGCCGACCGCFFGAIAAVLCCGLV  96
            LSSTP P P   A S  +   A     G   GA  G  FG +  VL  GL+
Sbjct  142  LSSTPTPGPNPTATSVSDPSTATDTGDGASAGAKAGIAFGILGGVLIVGLI  192


>ref|XP_010156447.1| PREDICTED: carboxypeptidase N subunit 2-like, partial [Eurypyga 
helias]
Length=575

 Score = 36.2 bits (82),  Expect = 2.8, Method: Composition-based stats.
 Identities = 22/61 (36%), Positives = 30/61 (49%), Gaps = 5/61 (8%)

Query  3    TAVSSTTYPAAAVTLDKKAMLPEVDIRREQPQVAP-----TMQSSSIPLSSTPVPSPPQV  57
            T  SST  P A++T     +LPE  I    P V P     T+  + +P +ST  P+ PQ 
Sbjct  505  TTPSSTVLPKASITTPSSIVLPETSIATLSPAVLPKAPITTLSPAMLPKTSTKTPTVPQE  564

Query  58   A  58
            A
Sbjct  565  A  565


>ref|XP_007702317.1| hypothetical protein COCSADRAFT_162491 [Bipolaris sorokiniana 
ND90Pr]
 gb|EMD61963.1| hypothetical protein COCSADRAFT_162491 [Bipolaris sorokiniana 
ND90Pr]
Length=1484

 Score = 35.8 bits (81),  Expect = 5.3, Method: Composition-based stats.
 Identities = 21/55 (38%), Positives = 31/55 (56%), Gaps = 9/55 (16%)

Query  11   PAAAVTLDKKAMLPEVDIRREQPQVAPTMQSSSI--PLSS-------TPVPSPPQ  56
            P     + +K  LP +DI + QPQ AP++QS++I  P SS       TP+ S P+
Sbjct  931  PGGRPIISRKPTLPLMDITQTQPQAAPSVQSTTILTPTSSIDDDMNKTPIQSNPE  985


>ref|XP_007688806.1| hypothetical protein COCMIDRAFT_97608 [Bipolaris oryzae ATCC 
44560]
 gb|EUC44685.1| hypothetical protein COCMIDRAFT_97608 [Bipolaris oryzae ATCC 
44560]
Length=1468

 Score = 35.4 bits (80),  Expect = 6.8, Method: Composition-based stats.
 Identities = 21/55 (38%), Positives = 31/55 (56%), Gaps = 9/55 (16%)

Query  11   PAAAVTLDKKAMLPEVDIRREQPQVAPTMQSSSI--PLSS-------TPVPSPPQ  56
            P     + +K  LP +DI + QPQ AP++QS++I  P SS       TP+ S P+
Sbjct  926  PGGRPIISRKPTLPLMDITQMQPQTAPSVQSTTILTPTSSIDDDMNKTPIQSHPE  980


>ref|WP_049326742.1| membrane protein [Rothia mucilaginosa]
Length=283

 Score = 34.7 bits (78),  Expect = 7.5, Method: Compositional matrix adjust.
 Identities = 26/71 (37%), Positives = 32/71 (45%), Gaps = 8/71 (11%)

Query  34   QVAPTMQSSSIPLSSTPVPSPPQVAQSTGEL--------EEAARIRGGGCCGACCGCFFG  85
            Q AP+ Q SS P+S+ PVP P  V  ++G L           A     G    C G FFG
Sbjct  103  QPAPSQQVSSQPVSAQPVPEPQWVPAASGYLTVRFVHTISALALPILAGVAVYCAGAFFG  162

Query  86   AIAAVLCCGLV  96
               A+   GLV
Sbjct  163  GPEALRIVGLV  173


>ref|XP_007710260.1| hypothetical protein COCCADRAFT_90853 [Bipolaris zeicola 26-R-13]
 gb|EUC35414.1| hypothetical protein COCCADRAFT_90853 [Bipolaris zeicola 26-R-13]
Length=1473

 Score = 35.0 bits (79),  Expect = 7.6, Method: Composition-based stats.
 Identities = 21/55 (38%), Positives = 31/55 (56%), Gaps = 9/55 (16%)

Query  11   PAAAVTLDKKAMLPEVDIRREQPQVAPTMQSSSI--PLSS-------TPVPSPPQ  56
            P     + +K  LP +DI + QPQ AP++QS++I  P SS       TP+ S P+
Sbjct  921  PGGRPIISRKPTLPLMDITQMQPQTAPSVQSTTILTPTSSIDDDMNKTPIQSHPE  975


>gb|KIW96195.1| hypothetical protein Z519_03263 [Cladophialophora bantiana CBS 
173.52]
Length=549

 Score = 35.0 bits (79),  Expect = 8.5, Method: Composition-based stats.
 Identities = 22/64 (34%), Positives = 31/64 (48%), Gaps = 0/64 (0%)

Query  3    TAVSSTTYPAAAVTLDKKAMLPEVDIRREQPQVAPTMQSSSIPLSSTPVPSPPQVAQSTG  62
            T  S     AAAV         E+D+ + +P  APT    S PLS T V +PP+  ++  
Sbjct  370  TLASYDVSAAAAVAGTVPEEHVELDMAQPEPHSAPTSPDGSPPLSPTAVATPPRPEENFP  429

Query  63   ELEE  66
             L+E
Sbjct  430  PLDE  433


Lambda      K        H        a         alpha
   0.325    0.135    0.421    0.792     4.96 

Gapped
Lambda      K        H        a         alpha    sigma
   0.267   0.0410    0.140     1.90     42.6     43.6 

Effective search space used: 656833657159


  Database: nr
    Posted date:  Sep 23, 2015 12:05 AM
  Number of letters in database: 26,053,659,533
  Number of sequences in database:  71,551,133


Matrix: BLOSUM62
Gap Penalties: Existence: 11, Extension: 1
Neighboring words threshold: 11
Window for multiple hits: 40
```
